# Misplaced subclavian central venous catheter

**DOI:** 10.11604/pamj.2017.27.59.9532

**Published:** 2017-05-26

**Authors:** Negussie Alula Bekele, Worknehe Agegnehu Abebe, Jemal Zeberga Shifa

**Affiliations:** 1 University of Botswana, Department of Anaesthesia and Critical Care Medicine, Botswana; 2 University of Botswana, Department of Surgery, Botswana

**Keywords:** Central venous catheter, misplacement

## Abstract

Percutaneous Central Venous Catheter (CVC) insertion using internal jugular and Subclavian veins routes is common procedure for all intensive care admitted patients and some patients in the ward as demand arises in central and referral hospitals of Botswana. This is a case report of a patient on whom a third attempt of re-inserting a CVC for fluid and total parenteral nutrition (TPN) was made. X-ray showed that left Subclavian inserted catheter was mis-directed to internal jugular vein of the same side creating discomfort to the patient. Ultra sound is recommended for routine investigation to confirm proper Central venous catheter placement as it can reduce failure, minimize complication and reduce cost of treatment.

## Introduction

This case presentation was an accidental finding of Misplaced CVC in a tertiary teaching hospital of Botswana. The hospital has a capacity of total of 500 beds for admission and with this there is 8 bedded multy disciplinary Intensive Care Unit (ICU) for intensive care service. The ICU accepts all patients from all the specialties except neonates as there is a neonatal ICU separately managed by paediatric intensive care specialists. The intensive care service is done by Anaesthesiologists and intensive care specialists whom are called to the wards to insert CVC as the need arises. Percutaneous Central venous catheter in our hospital is routinely inserted at time of admission to critically ill patients for the purpose of resuscitation fluid, administration of toxic drugs, long term hyper alimentation, measurement of central venous pressure, manage fluid administration and in emergency fluid volume restoration. This procedure is mainly done through two approaches where the most common are Infra-clavicular approach to subclavian vein and Internal Jugular vein access. The main reasons these routes are chosen is ease of insertion, comfort of the patient and ease of access for port of the catheter for administration of medications and fluids. Additionally it does not interfere the mobility of the patient. Possibly there are expected complications associated with insertion of CVC in these approaches where the main ones are pneumo/hydro-thorax, Malposition, Migration, introduction of infection and rarely failed procedure. The position of the tip of the catheter is expected to be in the Superior venacava (SCV) at the right atrial junction or there around.

## Patient and observation

**Figure 1:**
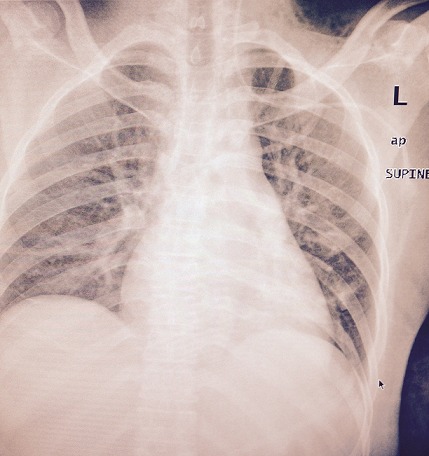
Patients CXR

**Figure 2:**
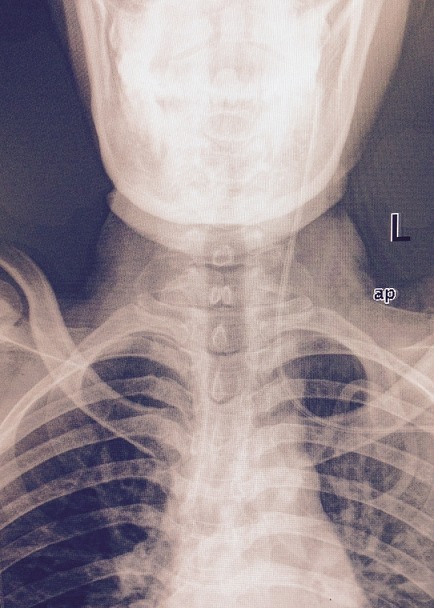
Patients Neck and Head X-ray

Our patient is a middle age woman diagnosed with intestinal obstruction, HIV positive on HAART had undergone surgery where resection of more than a meter small intestine was done. Five days post-surgery, she needed to have total parenteral nutritional therapy. Right side Internal Jugular vein was accessed, the CVC stayed only 2 days and then blocked. The second insertion of the CVC was done by another Anaesthesiologist using the right subclavian infraclavicular approach but soon was seen that this CVC was infiltrating and creating swelling around the insertion site. The third trial was done by a third Anaesthesiologist who was on call on that night and he inserted the CVC in the left Subclavian, infra clavicular approach at surgical ward. At time of insertion of the J guide wire the patient complained neck and ear pain at the side of catheter insertion for that the wire was removed and re-tried on the same spot. Catheter placement confirmed by drawing blood, putting the fluid bag below the heart level and back flow of venous non pulsating blood was observed, fluid flow initiated with room temperature normal saline but the patient still continued to complain discomfort at around the ear of the same side of insertion. Since the placement was sure but patient had pain with the flow of fluid, X-ray was ordered and result showed that the catheter was misplaced to the left internal Jugular vein and was found to be deep inserted. Catheter pulled 3 cm out and checked if it was still in position and then fluid administration and TPN continued with no problem. After the CVC insertion the patient was followed in our hospital and there were no complication noted (Figure 1,Figure 2).

## Discussion

CVCs are inserted for different indications such as administering drugs, monitoring central venous pressure (CVP), measurement of central venous oxygen saturation (ScvO2), renal replacement therapy, total parenteral nutrition, poor peripheral venous access, cardiac catheterization, and Trans venous cardiac pacing. Insertion done by different healthcare professionals at different sites of the hospital locations and with varying techniques for insertion and for ensuring correct catheter placement. The same holds true to PMH and the indication for this patient was post operation fluid administration and Total Parenteral Nutrition initiation. A thoracic surgeon and Anaesthesia professors Tae-Eun Jung and Daelim Jee, on journal of clinical anaesthesia reported a Misplaced central venous catheter in the jugular venous arch exposed during dissection before sternotomy [1]. The ideal catheter tip position is widely accepted that the tip of the catheter should be in as large a central vein as possible typically the superior vena cava (SVC) or inferior vena cava (IVC), ideally outside of the pericardial sac, and parallel with the long axis of the vein. There are potential complications associated with all tip positions. The most proximal venous valves lie 2.5 cm from the termination of the internal jugular (IJ) and subclavian veins (SCVs). Incorrect catheter placement proximal to these valves will lead to inaccurate CVP monitoring or potential irritation of the valve area by the catheter or infused fluids [1,2]. Xiaoyan Gu and colleagues reported a case in which a CVC was inadvertently directly inserted into the right main pulmonary artery, and its malposition was detected by transesophageal echocardiography (TEE) [2]. Positioning of the tip of the catheter in unintended place is relatively common estimated to be 5.4% from SCV to the IJV, no difference whether insertion is in the right or left side [3]. Catheter tip placements in other directions like in the contralateral IJV through left innominate vein, left internal mammary vein, azygous vein, hemiazygos vein, lateral thoracic vein, inferior thyroid vein, left superior intercostal vein, thymic vein, ipsilateral arm vein, and the jugular foramen have been also reported [4]. ″A report of a case showed malposition of a central venous catheter (CVC) in a partial anomalous left-sided pulmonary vein draining into a left innominate vein″ [4]. Lewis A. Eisen, analysed 385 consecutive central venous catheter (CVC) attempts over a 6-month period on adult patients. The rate of mechanical complications not including failure to place was 14% and improper position was 14 out of 258 subclavian and internal jugular attempts of CVC insertions [5]. Mechanical complications are reported to occur in 5 to 19 percent of patients, infectious complications in 5 to 26 percent, and thrombotic complications in 2 to 26 percent. It was noted association of malposition and thrombo embolic complications in a study done by Alain Luciani and colleagues, on 145 patients it was found that 17 patients had developed thrombo embolic complications and out of these 17 patients 13 was related to incorrect placement of tip of catheter [6].

Tiberiu Ezri and etal discussed that with many of the currently described positioning methods, malposition of a CVC is frequent, leading in extreme cases to life-threatening complications. Some clinicians consider right atrial electrocardiography- assisted CVC insertion However; it may fail to differentiate between placement in the superior vena cava (SVC) and the right atrium (RA). Special CVCs equipped with electrocardiographic electrodes are expensive and not always available. Trans oesophageal echocardiography could be good but the apparatus and the performance skills are also not always available. The author based on these explanation tried to recommend the advantage of use topographic CVC positioning technique which is commonly used in our institutions but still did not confirm sufficient guarantee for the accuracy of proper placement of the catheter. we believe that the most suitable and affordable instrument with a big yield of good result could be ultra sound guided CVC insertion [7,8]. Our idea is well supported by Andrew Bowdle on a review article on Barash text book of Anaesthesia advocating for the relatively inexpensive, portable ultrasound equipment for CVC placement in order to reduce failure and complications [9]. Eric Maury and colleagues evaluated ultrasonic examination as a diagnostic tool for catheter misplacement and pneumothorax after central venous catheter insertion. The results were compared with those of chest radiography. Eighty-five central venous catheters (70 subclavian and 15 internal jugular) were inserted into 81 patients; 10 misplacements and one pneumothorax occurred. Ultrasonic examination feasibility was 99.6%. The only pneumothorax and all misplacements except one were diagnosed by ultrasound [10]. F. Gibson and A. Bodenham published on BJA in February 5, 2013 nothing the great increase of successful proper placement of CVC by using Ultrasound, ECG guidance, real-time X-ray imaging, and other aids [11]. This finding by itself showed that there is a need of assessment of inserted CVC catheter is necessary and US is a mobile instrument that can be used immediately after the insertion and it can reduce the exposure to radiation,. On a discussion of pro and cons of UG-CVC insertion by John G.T. Augoustides and colleagues noted that the advent of UG-CVC significantly improved vein localization, cannulation success, and freedom from complications. It was also noted that it is cost effective even though it demands training of personnel and purchasing of equipment [12, 13]. In a recent expert recommendation on safety practices for vascular access. UG-CVC has already been proposed as a standard of care by national medical agencies such as the IOM, AHRQ, and NICE for more than 5 years [13]. In our hospital X-ray examination is used to confirm CVC placement but all patients are not examined after insertion of Catheter. Our case report showed that the left side inserted subclavian catheter unintentionally has been directed itself in the left internal Jugular vein and has created discomfort repeatedly. This could have been detected by routine use of X-Ray and preferably ultrasound after CVC insertion.

## Conclusion

This case shows that there is a risk of failure to pick misplacement of CVC. We recommend routine use of ultra sound guided insertion of CVC and training of Ultra sound guided insertion of CVC. Even though further study is required, it is obvious that use of ultra sound will reduce failure, Complications, outcome and cost of treatment of patient.

## Competing interests

The authors declare no competing interest.
